# The Financial Situation

**Published:** 1914-12

**Authors:** 


### The Financial Situation

Six years ago, a period of financial depression set in, resulting in many failures. With perhaps a year's remission, this country has had hard times ever since. For a period of nearly a year and a half, there was an average of one failure or discontinuance of business a month, in a single block on Main street, Buffalo. In spite of these conditions, the BUFFALO MEDICAL JOURNAL has prospered, mainly, of course, by increasing its advertising, though this could not have been accomplished except by increasing the circulation. Correspondingly, the expenses have increased in spite of rigorous economy. As compared with 1911, the volume contains more than the equivalent in bulk, of 13 issues and, by condensation in various departments, and exclusion of matter not of interest except in a historic and, perhaps, sentimental way, the actual medical material contained in the volume is at least 50% greater. Illustrations have been used more freely and, with the gradual increase in cost of paper, printing, etc., it has been neither possible nor desirable to accumulate a surplus beyond a small amount as a margin of safety. Barring an inevitable and comparatively small loss on advertising accounts, this portion of the receipts of the Journal is satisfactory. The majority of the subscribers have also shown a commendable spirit of co-operation in various ways, including the very practical one of remitting promptly. There are, however, enough two-dollar units, multiplied by the number of subscribers in arrears and, in some instances, by quite a number of years, to total a sum sufficient to print the Journal for about three issues. The lack of this sum, especially under existing conditions of general financial stringency, requires time and effort which we think might better be expended on the reading matter of the Journal.

